# Long-term benefits for lower socioeconomic groups by improving bowel screening participation in South Australia: A modelling study

**DOI:** 10.1371/journal.pone.0279177

**Published:** 2022-12-21

**Authors:** Anita Lal, Lan Gao, Elise Tan, Nikki McCaffrey, David Roder, Elizabeth Buckley

**Affiliations:** 1 Deakin Health Economics, Institute for Health Transformation, Deakin University, Geelong, Victoria, Australia; 2 Cancer Council Victoria, Melbourne, Victoria, Australia; 3 Cancer Epidemiology and Population Health Research Group, Allied Health and Human Performance Academic Unit, University of South Australia, Adelaide, South Australia, Australia; 4 Flinders Health and Medical Research Institute, Flinders University, Adelaide, South Australia, Australia; LSU Health Sciences Center New Orleans: Louisiana State University Health Sciences Center, UNITED STATES

## Abstract

**Introduction:**

The gap in bowel cancer screening participation rates between the lowest socioeconomic position (SEP) groups and the highest in Australia is widening. This study estimates the long-term health impacts and healthcare costs at current colorectal cancer (CRC) screening participation rates by SEP in South Australia (SA).

**Methods:**

A Markov microsimulation model for each socioeconomic quintile in SA estimated health outcomes over the lifetime of a population aged 50–74 years (total n = 513,000). The model simulated the development of CRC, considering participation rates in the National Bowel Cancer Screening Program and estimated numbers of cases of CRC, CRC deaths, adenomas detected, mean costs of screening and treatment, and quality adjusted life years. Screened status, stage of diagnosis and survival were obtained for patients diagnosed with CRC in 2006–2013 using data linked to the SA Cancer Registry.

**Results:**

We predict 10915 cases of CRC (95%CI: 8017─13812) in the lowest quintile (Q1), 17% more than the highest quintile (Q5) and 3265 CRC deaths (95%CI: 2120─4410) in Q1, 24% more than Q5. Average costs per person, were 29% higher in Q1 at $11997 ($8754─$15240) compared to Q5 $9281 ($6555─$12007). When substituting Q1 screening and diagnostic testing rates with Q5’s, 17% more colonoscopies occur and adenomas and cancers detected increase by 102% in Q1.

**Conclusion:**

Inequalities were evident in CRC cases and deaths, as well as adenomas and cancers that could be detected earlier. Implementing programs to increase screening uptake and follow-up tests for lower socioeconomic groups is critical to improve the health of these priority population groups.

## Introduction

Colorectal cancer (CRC) has the second-highest disease burden of cancers in Australia [1] and the highest health system expenditure of cancers at $1.1billion [2]. Australians living in the most disadvantaged areas of Australia have the highest incidence rate of CRC (61 compared to 52 per 100,000 for the most advantaged) and the lowest 5-year observed survival (57% compared to 63% for the most advantaged) [3]. Early detection using faecal occult blood test (FOBT) screening significantly improves CRC survival outcomes [4]. The National Bowel Cancer Screening Program (NBSCP) was introduced in Australia in 2006. Age-specific participation rates of screening have been increasing however, Australians from the lowest socioeconomic group had a lower participation rate (40%) compared to those in the highest group (45%) in 2019 [7].

The NBSCP includes people aged 50–74 who are invited biennially to participate and is provided free of charge under Medicare, Australia’s universal health insurance scheme. Medicare is funded by the Commonwealth Government through a taxpayers’ levy and all Australian citizens and permanent residents are eligible. Bowel cancer screening kits are delivered to peoples’ homes, are self-administered, include a postage paid return envelope and participants do not have to see a health professional.

In countries with universal healthcare such as Australia, the UK and Canada, financial barriers to screening are less likely to play a role, however socioeconomic inequalities in screening participation are evident [9, 10]. Todorov and colleagues [5] identified one of the main reasons reported for differences in participation by lower socioeconomic groups in South Australia was the lack of understanding that screening is effective when no symptoms are present. Limited health literacy (i.e. a low capacity to obtain, process, understand and use healthinformation) disproportionately affects people from lower socioeconomic positions [6]. Limited health literacy is a significant barrier to bowel screening that influences screening knowledge, beliefs and behaviour [7].

A main aim of government screening strategies in Australia is improving equity of access for disadvantaged groups [8]. Studies from Australia that have modelled the long-term health outcomes and cost-effectiveness of the NBCSP have focused on the whole population without an equity lens. At current participation rates the NBCSP is underutilised and substantial additional investment could be spent on improving participation while still remaining cost-effective [9]. The estimated maximum spending levels of effective interventions to improve screening rates from 40% to 60%, was estimated to be AUD$214–$502 per person and AUD$44–$91 per person to improve the diagnostic assessment rate (colonoscopy following positive test) [16].

Variation in CRC outcomes could be reduced if screening participation improved in priority population groups. This primary aim of this study is to determine the long-term health and healthcare costs disparities of CRC across socioeconomic groups in SA. The eligible screening population of the NBCSP will be examined from a healthcare perspective. The findings will provide evidence of long-term equity impacts for disadvantaged groups and whether enhancements to boost program outcomes for the less screened populations should be implemented, thereby improving health equity. The outcomes of this study will also inform the National Bowel Cancer Screening Program.

## Methods

### National Bowel Cancer Screening program

#### Intervention description

In 2019, residents of Australia aged 50–74 enrolled in Medicare, were invited biennially to participate in the NBSCP via mail. Therefore, the NBCSP invited Australians aged 50, 52, 54, 55, 58, 60, 62, 64, 66, 68, 70, 72 and 74 to participate. The eligible population are sent an FOBT kit, an information booklet and a reply-paid envelope. The test involves collecting two separate samples of faeces. After completing the test, participants mail the samples to the NBCSP pathology laboratory for analysis. The test is free of charge for participants.

### Study population

The simulated population of 513,000 is equal to the estimated 2019 South Australian resident population aged 50 to 74 years [10]. The model assumes there are 102,600 people in each Socioeconomic Index for Areas (SEIFA) quintile. The SEIFA quintiles represent groups of individuals who live in similarly ranked areas, based on a range of information such as the income, qualifications, and occupation skills of the area residents [11]. Proportions of the population in each age group were sourced from the Australian Bureau of Statistics (ABS) and were assumed to be the same for each quintile [12]. (S1 Table) The microsimulation model walks the same cohort of 102,600 people through each SEIFA quintile arm of the model over a 50 year time horizon.

### Health benefit modelling

#### Overview

We developed the Priority Population Microsimulation Colorectal Cancer (PRISM-CRC) model, which consists of five identically structured markov nodes to estimate the lifetime costs and benefits of the NBSCP by SEIFA quintile. The base case estimates the health outcomes–cases of CRC, mean quality adjusted life years (QALYs) gained, deaths and mean healthcare costs from current screening rates by SEIFA quintile. The second analysis estimates the health outcomes if screening rates of the more disadvantaged quintiles are replaced with those of the most advantaged quintile to mimic the addition of a targeted screening intervention aimed at lower SEIFA quintiles. The simulated cohort moves through the Markov model in 1-year cycles until death. At the end of each 1-year period the individual person either moves to a different health state or stays in the current health state. The model consists of three parts 1) natural cancer development 2) screening and 3) colonoscopy surveillance. Participants start in either screening or natural cancer development. A summary of the key input parameters used by Priority Population Microsimulation Colorectal Cancer (PRISM-CRC) model is provided in Table 1.

**Table 1:** Key model parameters used by the Priority Population Microsimulation Colorectal Cancer (PRISM-CRC) model.

Model Parameter			Value (uncertainty range)				Source & notes
**CRC incidence by SEIFA quintile 2010–2014 and rate ratios**			**Incidence/ 100,000**		**Rate ratio**		
1			63.3		1.07		Cancer Australia [25]. Rate ratio calculated using the Aus. population average as the denominator
2			62.2		1.05		
3			59.1		1.00		
4			57.70		0.97		
5			53.3		0.90		
Population average			59.8				
**2017 population incidence of CRC**							
50–54 years			0.0006				SA Cancer Registry [13]
55–59 years			0.0008				
60–64 years			0.0013				
65–69 years			0.0017				
70–74 years			0.0023				
75–79 years			0.0028				
80–84 years			0.0038				
85+ years			0.0034				
**Population Incidence of progressive adenomas, age adjusted**							
50–54			0.0023				Calculated as 20 years prior to CRC incidence [14, 15] ie age 50–54 is incidence of cancer age 70–74.
55–59			0.0028				
60–64			0.0038				
65+			0.0034				
**Incidence of all adenomas**							
50–54			0.0095				Incidence of adenoma adjusted for proportion of all adenomas that develop into progressive adenomas of 24% [16]
55–59			0.0117				
60–64			0.0158				
65+			0.0142				
**Incidence per person low risk adenomas**							
50–54			0.0072				Incidence of all adenomas minus Incidence of progressive adenomas
55–59			0.0089				
60–64			0.0120				
65+			0.0108				
**Transition probability**							
Low risk to high risk			0.02 (0.01–0.04)				Frazier et al [30]
High risk to CRC			0.05 (0.02–0.10)				
Stage A to Stage B			0.05 (0.02–0.10				
Stage C to Stage D			0.28 (0.10–0.50)				
Low risk to high risk			0.02 (0.01–0.04)				
**Disease prevalence from colonoscopy assessment**			**Value**				
Free of disease			53.7%				AIHW 2019 [17]
Polyps or Low risk adenoma			37.7%				
High risk adenoma			5.8%				
CRC present			2.7%				
**Distribution of CRC by stage and screening status**			**Screened**	**Not Screened**			
Stage A			28.6%	16.0%			SA linked data set
Stage B			23.8%	29.3%			
Stage C			37.2%	32.0%			
Stage D			10.4%	22.7%			
**Screening Participation rates by SEIFA quintile SA 2017–2018**							Calculated from AIHW[18] and ABS [11]
1			41.9				
2			47.6				
3			48.1				
4			51.3				
5			51.7				
**Colonoscopy assessment rate**							AIHW 2020 [19]
Q1			59.4				
Q2			59.5				
Q3			66.0				
Q4			71.7				
Q5			74.3				
Screening positivity rate							AIHW 2020 [19]
Q1			7.9				
Q2			7.1				
Q3			6.7				
Q4			6.3				
Q5			5.4				
**Probability of CRC diagnosis if not screened**			**Diagnosis probability**	**Range**		**Calculation**	Calculations based on distribution of CRC by stage, not screened (from cells above)
Stage A			0.16	0.06–0.26		row 1	
Stage B			0.35	0.25–0.45		row 2/(1 –row 1)	
Stage C			0.59	0.49–0.69		row 3/(1 –row 1 –row 2)	
Stage D			1.000	0.90–1		row 4/(1 –row 1 –row 2 –row 3)	
**Survival by stage 1–5 years**							
**Dukes Stage**	**Year 1 (95% CI)**	**Year 2 (95% CI)**	**Year 3 (95% CI)**	**Year 4 (95% CI)**		**Year 5 (95% CI)**	SA linked dataset
Stage 1	0.97 (0.96, 0.9)	0.97 (0.95, 0.98)	0.96 [0.94, 0.97)	0.95 (0.93, 0.97)		0.94 (0.92, 0.96)	
Stage 2	0.96 (0.95, 0.97)	0.93 (0.91, 0.94)	0.89 (0.87, 0.90)	0.87 (0.84, 0.89)		0.84 (0.82, 0.87)	
Stage 3	0.92 [0.90, 0.93)	0.83 (0.80, 0.85)	0.76 (0.73, 0.78)	0.70 (0.67, 0.73)		0.67 (0.64, 0.69)	
Stage 4	0.61 [0.58, 0.64)	0.40 (0.37, 0.43)	0.25 (0.22, 0.28)	0.19 (0.16, 0.22)		0.15 (0.12, 0.19)	
**FOBT costs**			Value (range)				
FOBT kit			$8.00 (10–12)				Assumption based on previous Australian study [14]
Packaging and postage			$2.40				Two padded envelopes $0.10 each [20], $1.10 return postage 250g [21]
Test analysis in laboratory			$17.85				MBS item 66767, Department of Health [22]
**Colonoscopy**							
General Practitioner consultation			$37.60				MBS item 237, Department of Health [22]
Colonoscopy, no biopsy (without complication)			$2258				AR-DRG item G48B, Independent Hospital Pricing Authority [23] inflated to 2019
Cost of colonoscopy with polypectomy			$4203				AR-DRG item G47B, Independent Hospital Pricing Authority [23] inflated to 2019
**CRC Treatment**							
Stage A			$40,999 (37,657–46,025)				Mean costs inflated to 2019 dollars, Ananda et al [24] AIHW [25]
Stage B			$52,594 (48,307–59,041)				
Stage C			$95,754 (87,948–107,492)				
Stage D			$90,272 (82,913–101,338)				
Discount rate			5%				Australian Government Department of Health and Aged Care [26]

Notes: SEIFA; Socioeconomic Index for Areas; FOBT: faecal occult blood test; MBS: Medicare Benefits Schedule; AR-DRG: Australian Refined Diagnostic related group.

#### Colorectal cancer model

The first component of the modelling is the natural development of CRC, which influences the movement of patients to various health states. There are seven health states within the model

1. Free of disease
2. Low risk adenoma
3. High risk adenoma
4. Stage A CRC
5. Stage B CRC
6. Stage C CRC
7. Stage D CRC

The stage of cancer is classified according to Australian Clinico-Pathological Staging (ACPS) system from A to D and indicates how far the cancer has spread anatomically [27]. Similar to the American Joint Committee on Cancer (AJCC) staging system, there are four main stages. Stage A of the ACPS translates to American Joint Committee on Cancer (AJCC) Stage grouping I, Stage B translates to IIA-IIC, Stage C translates to IIIA-IIIC and Stage D translates to IVA-IVC [28]. The model categorises the stage as either diagnosed or undiagnosed. In the non-screened group,diagnosed cancer will occur when a patient presents with symptoms (i.e., diagnosed at a more advanced stage compared to screened). The transition probabilities from one state to another attempt to capture the natural history of cancer. Life expectancy of the patient depends on the five-year stage-specific survival figures. Participants who are free of disease or have non-progressive adenoma for five years or more can die of other deaths determined by background mortality rates.

There are several assumptions in the model. The underlying risk of CRC remains constant using SA population CRC incidence rates in 2017. Patients who survive more than five years after diagnosis are assumed to be cancer survivors and have general population mortality rates with no recurrent CRC. The model does not account for the people with increased risk of CRC due to family history and assumes a maximum age of 100 years.

#### CRC screening

**Fig 1:**
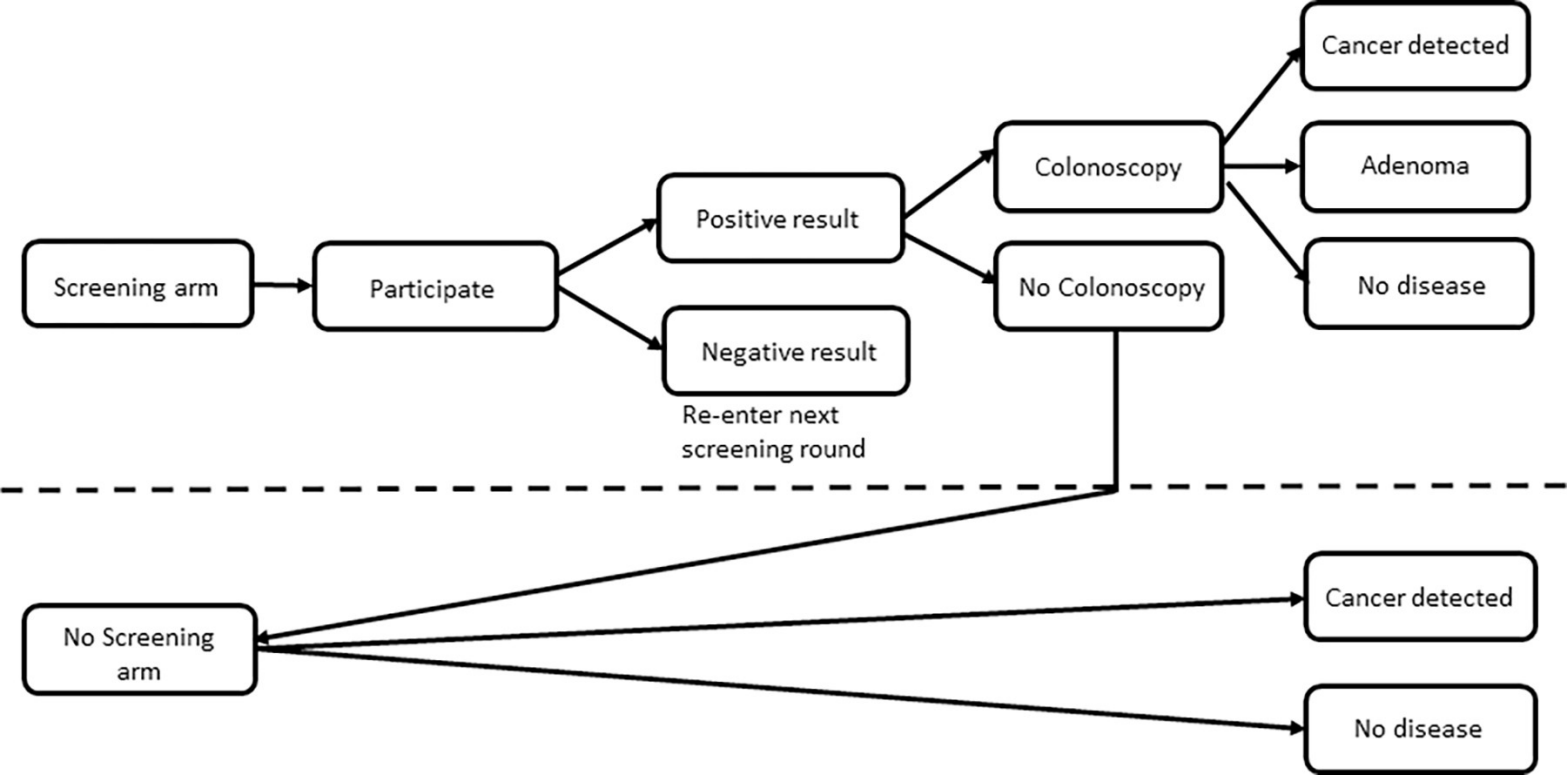
Screening and outcomes pathway.

The screening pathway is illustrated in Fig 1. Screening aims to disrupt the natural progression of CRC to prevent CRC or detect CRC early and increase life expectancy. The model estimates screening and diagnostic pathways based on participation rates, screening test results and adenoma and CRC incidence. In the screened group adenomas and non-symptomatic cancer may be diagnosed by FOBT test and colonoscopy. The eligible aged population are invited to be screened and if they participate, they receive either a positive or negative FOBT. The model assumes that all FOBT tests are completed correctly. If the result is negative, participants are invited to screen again in the next round. If the result is positive, participants either consult a general practitioner to obtain a referral for colonoscopy for further diagnosis or take no action. Those who take no action after a positive FOBT are moved to the natural cancer development arm and are assumed to be diagnosed without screening.

Markov models were used to track patients in the SA population aged 50 to 74 through the NBSCP from the point of participation in screening to death or aged 100 years. People without CRC may undergo multiple sceenings during the simulated time horizon until they reach age 75, unless they have died earlier due to other causes. S1 Appendix describes the PRISM-CRC model in detail.

*Screening participation rates*. To calculate participation rates by SEP, participation rates for South Australian residents by statistical area level 2 (SA2) as classified by the ABS, which align with one or more related suburbs [18] were matched with the ABS, Index of Relative Socioeconomic Disadvantage, Socioeconomic Index for Areas (SEIFA) deciles [29]. SEIFA deciles represent groups of individuals who live in similarly ranked areas, based on a range of information such as the income, qualifications, and occupation skills of the residents. SEIFA deciles were combined to create quintiles

#### Data sources

A linked data platform was used for all patients in SA diagnosed with CRC (ICD10 18–20) as recorded by the South Australian Cancer Registry (SACR). Cancer patient records were linked to the NBCSP through the Australian Instutute of Health and Welfare (AIHW) and the National Death Index. This analysis uses patient records from 1 January 2006 to 31 December 2013.

#### Diagnostic variables

Positivity rates of FOBT screening and subsequent colonoscopy rates by age and SEIFA observed in 2018 for Australia from the AIHW NBCSP monitoring report were used [19]. We then used the reported diagnostic results from the colonscopies due to the program, to calculate the probabilities of adenoma, cancer and no disease in the model [24].

#### Clinical variables

Details of the main clinical variables used in the modelling are displayed in Table 1. The prevalence of adenoma and CRC for the unscreened population was estimated using SA population CRC incidence rates in 2017 for the lifetime of the model [13]. Incidence of adenoma and progressive adenoma was calculated using the age specific incidence of CRC and taken back to when an adenoma is likely to have developed. The time from progressive adenoma incidence to cancer diagnosis is assumed to be 21 years, based on an average from previous CRC modelled studies [14, 15]. The estimated proportion that develop into malignancies is 24% [16]. Progressive adenoma incidence was subtracted from all adenoma incidence to obtain the low risk adenoma incidence [16]. As there are no published quintile specific incidence rates by age, rate ratios based on SEIFA quintile and population incidence rates were calculated and used to adjust the population CRC incidence rates by age. For the screened population, adenoma and cancer prevalence data from colonoscopy assessment outcomes reported by the AIHW [17] were used. At diagnosis, the proportions in each stage of CRC by screening status were extracted from the SA linked dataset.

Once the participant has entered the model, they move to another health state based on the probability of progression, calculated on a yearly basis. For the unscreened population, the natural history of adenomas was reproduced using three transition probabilities: adenoma incidence, transition from low to high-risk adenoma and transition from high-risk adenoma to CRC. Transition probabilities from low to high-risk and high-risk adenoma to CRC and CRC stage were based on Frazier et al [30] which have been estimated by calibration to U.S cancer incidence and stage distribution. Probability of diagnosis at each stage was estimated based on the percentage of people at each stage of cancer in the unscreened population using the methods reported in a previous CRC modelling study [31]

#### Survival

When a participant is diagnosed with CRC, they either remain in that health state (cancer stage), progress to a more advanced cancer stage or if they survive more than 5 years they move to a ‘recovered’ health state. The life expectancy is calculated based on 5-year survival rates extracted by stage from the SA linked data set. If a participant survives more than 5 years, they are assumed to have the same life expectancy as the average population. Non-CRC mortality by age was obtained by subtracting the CRC mortality rate in SA 2016 [32] from the all-cause mortality rate in Australia 2016–2018 [33].

#### Outcomes

Quality adjusted life years (QALYs) were used as a measure of any health gains associated with the NBCSP. QALYs are calculated by multiplying the utility weight associated with a health state (represents quality of life) by the length of life lived in that health state (represents quantity of life) [34, 35]. Utility-weights are measured on a scale from 0 to 1, in which ‘0’ represents being dead and ‘1’ refers to perfect health. In this study, utility scores based on a meta-analysis by CRC stage were used, with stages A to C valued at 0.74 and stage D valued at 0.68 [36]. Australian population norm utility scores, for the population without CRC were valued at 0.80 for ages 50–69, 0.76 for ages 70–79 and 0.70 for 80 years and over [37]. QALYs were discounted at a rate of 5%.

### Healthcare costs

The reference year for costs is 2019. The FOBT screening costs include the FOBT kit, return postage and laboratory processing for Medicare Benefits schedule (MBS) item 66767. If a patient needs a colonoscopy they are referred to a general practitioner for a consultation costed as MBS item 23 [22]. The cost of colonoscopy procedure was assigned based on whether a polypectomy was performed and whether complications occurred. Treatment costs were based on CRC stage taken from an Australian study [24] that looked at cost of cancer therapies. Half the patients in Stage 4 receiving chemotherapy also received bevacizumab medication at a cost of approximately $10,000, therefore an average of the cost of treatment with and without bevacizumab was used. Costs were inflated to 2019 using the AIHW total health price index [25]. A discount rate of 5% was applied to costs.

### Uncertainty and sensitivity analyses

Uncertainty analyses were undertaken to evaluate how robust the results are to the assumptions made in the analysis. A probabilistic multivariate uncertainty analysis was conducted using Monte Carlo simulation method. Means and 95% uncertainty intervals for incremental costs, are reported based on 2000 iterations of the model using TreeAge software. Uncertainty parameters are presented in Table 1.

We also ran the model with a start age of 50 over a 35 year period to validate the results with CRC incident cases and deaths from the South Australian Cancer Registry in 2017 [13].

## Results

Within the context of the National Cancer Bowel screening program for the South Australian population aged 50–74, 10915 (95%CI: 8017─13812) cases of CRC are predicted in the lowest quintile Q1, 2580 (31.0%) more cases compared to the highest (Q5) (Table 2). In terms of mortality, when Q5 is compared to the lower quintiles, 692 (26.9%) more CRC deaths occur in Q1 and 609 (23.7%) more deaths in Q2. One of the largest differences is the estimated number of colonoscopies not taken up following a positive FOBT test in the lower quintiles, 1232 (80.2%) fewer colonoscopies in Q2 than Q5 and 1073 (69.8%) fewer colonoscopies in Q1 than Q5. Further, there were 29.7% and 12.7% fewer adenomas and CRCs detected by screening in Q1 and Q2 respectively compared to Q5.

**Table 2:** Modelled cohort analysis of the health outcomes over the lifetime and substituting screening participation and follow up colonoscopy rates for Q1 and Q2 with Q5 rates.

	Q1	Q2	Q3	Q4	Q5*	Q1 with Q5	Q2 with Q5
**CRC cases, % Difference to Q5 (95% CI)**	10915 (8017─13812)	10584 (7759─13482)	9790 (7116─2688)	9235 (6676─12133)	8335 (5981─11232)	10449 (7600─13298)	10197 (7400─12995)
	31.0% (23.0%−34.0%)	27.0% (20.0%−29.7%)	17.5% (13.0%−19.0%)	10.8% (8.0%−11.6%)		25.4% (-8.8%─59.5%)	22.3% (-11.2%─55.9%)
**Screen detected adenoma or CRC % Difference to Q5 (95% CI),**	438 (28─848)	544 (91─998)	591 (115─1067)	661 (166─1155)	623 (138─1109)	883 (300─1466)	812 (300─1323)
	−29.7% (−23.5%−−79.5%)	−12.7% (−10.0%−−34.4%)	−5.2% (−3.8%—16.6%)	6.0% (−4.2%—20.1%)		41.6% (-51.9%─135.1%)	30.2% (-1.9%─112.3%)
**CRC deaths, % Difference to Q5 (95% CI)**	3265 (2120─4410)	3182 (2056─4327)	2968 (1889─4113)	2814 (1762─3959)	2573 (1563─3718)	3151 (2100─ 4202)	3076 (2000─4152)
	26.9% (18.6%─35.6%)	23.7% (16.4%─31.5%)	15.4% (10.6%─20.9%)	9.4% (6.5%─12.7%)		22.5% (-18.4%─63.3%)	19.5% (-22.3%─61.3%)
**No follow-up colonoscopy, % Difference to Q5 (95% CI)**	2610 (1618─3602)	2769 (1759─3779)	2278 (1360─3195)	2075 (1183─2966)	1537 (769─2305)	2165 (1300─3030)	1990 (1200─2780)
	69.8% (56.3%─110.4%)	80.2% (64.0%─128.7%)	48.2% (38.6%─76.8%)	35.0% (28.7%─53.8%)		40.9% (-15.4%─97.1%)	29.5% (-21.9%─80.8%)
**Average cost per person, % Difference to Q5 (95% CI)**	$11997 ($8,754─$15,240)	$11652 ($8429─$14876)	$10823 $7765─$13881)	$10254 ($7316─$13191)	$9281 ($6555-$12007)	11594 (8459─14730)	11289 (8092─14486)
	29.3% (26.9%─33.5%)	25.6% (23.9%─28.6%)	16.6% (15.6%─18.5%)	10.5% (9.9%─11.6%)		24.9% (-8.9%─58.7%)	21.6% (-12.8%─56.1%)
**QALYs per person % Difference to Q5 (95% CI)**	12.86 (12.59─13.12)	12.88 (12.62─13.14)	12.89 (12.63─13.15)	12.91 (12.65─13.16)	12.92 (12.66─13.18)	12.88 (12.62─13.14)	12.89 (12.62─13.15)
	−0.50% (0.46%−0.55%)	−0.35% (0.33%−0.37%)	−0.26% (0.25−0.28%)	−0.122% (0.120%−0.124%)		-0.32% (-2.33%─1.70%)	-0.27% (-2.31%─1.78%)

Notes: *Reference socioecomic quintile (most advantaged), cost includes sreening, diagnosis and treatment, CI; confidence intervals, CRC; colorectal cancer. The Markov cohort model employed parameters in which uncertainty was not evaluated at the individual level, therefore no p-values can be supplied for the quintile differences.

Average costs per person, were over 25% higher in the two most disadvantaged quintiles, reflecting more cases and later stage treatment costs in these groups. Average quality adjusted life years (QALYs) per person, were slightly lower in the most disadvantaged quintiles with differences of 0.06 and 0.04, around 0.05% less compared to the most advantaged quintile. In a quintile population of 102,600 people, this equates to 6156 and 4104 QALYs respectively.

**Fig 2:**
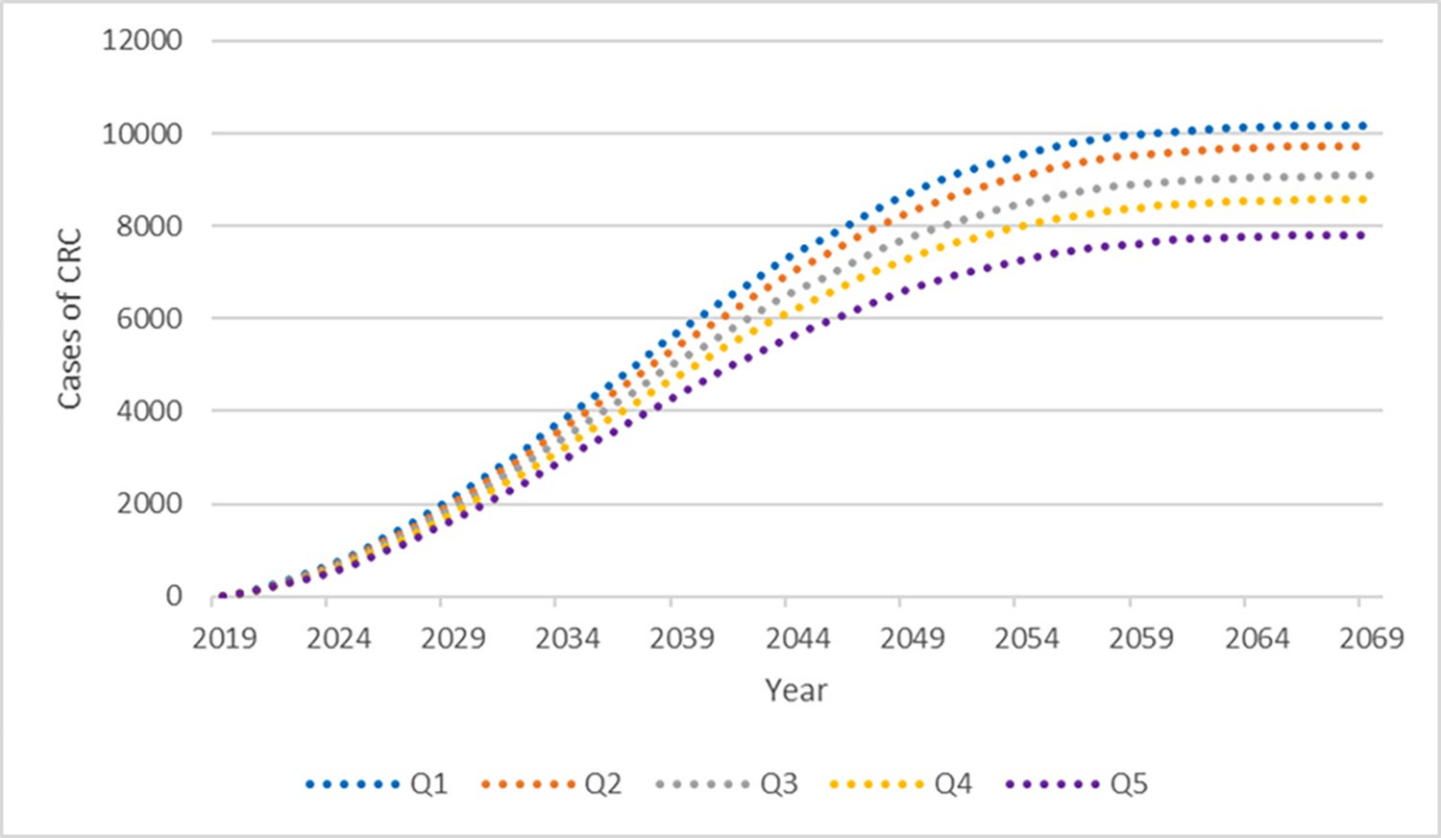
Predicted cases of CRC aged 50–74 years over the period 2019–2059 by SEIFA quintile.

**Fig 3:**
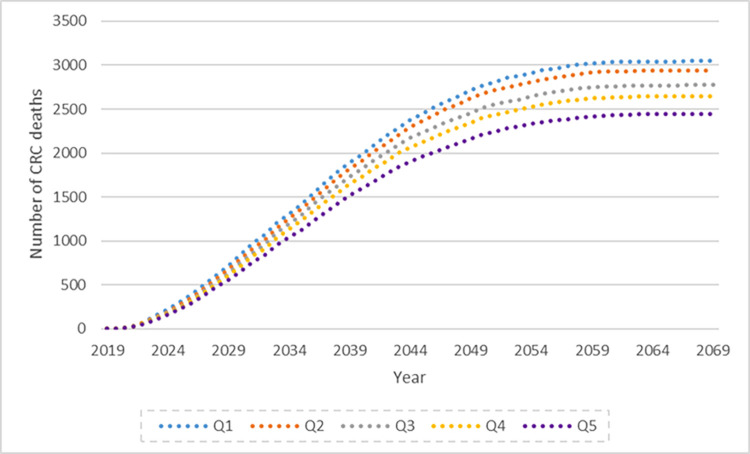
Predicted CRC deaths aged 50–74 years over the period 2019–2059 by SEIFA quintile.

Figs 2 and 3 illustrate the predicted cases of CRC and deaths from CRC over the 50-year period for the cohort aged 50 to 74 starting in 2019. The SEIFA quintile gradient is apparent, with higher cases and deaths in the lower quintiles and a larger difference between Q5 and the lower quintiles.

When substituting the screening participation and follow up colonoscopy rates for Q1 and Q2 with Q5 rates, adenoma or cancers detected increased by 445 (101.5%) in Q1 and 268 (49.2%) in Q2. The percentage difference of cancer cases reduced by 466 (4.3%) and 387 (3.7%) in Q1 and Q2 respectively. Follow up colonoscopies after a positive result increased by 445 (17.0%) and 779 (28.1%) in Q1 and Q2 respectively.

### Model validation

The model predicts 48,859 CRC cases over 50 years, i.e. 977 per year for the cohort of 513,000, slightly lower than the most recently reported new CRC cases in 2017 of 1078 in SA for persons aged 50 and over [22]. This difference is likely due to the lower number of people aged 50–74 at the beginning of the 50-year period in the fixed, modelled cohort and the additional new cases (approximately 150) reported in the ages outside the screening age groups in the ages of 75–79, 80–84 and 85 and over in the SA population [22].

### Sensitivity analysis

The reported cases of CRC deaths in 2017 from the South Australian Cancer Registry was 387 with 118 deaths occurring at age 85 and over [22]. The model predicts a total of 14,801 CRC deaths over 50 years, or an average 296 deaths per year, lower than the predicted deaths, due to the cohort size. However, when using a start age of 50 years and a time horizon of 35 years, 13,961 deaths are predicted or 399 deaths per year which is close to the 387 reported deaths in 2017 by the South Australian Cancer Registry [22].

## Discussion

We found that at current levels of bowel cancer screening by socioeconomic quintile in SA for the cohort aged 50 to 74 years, there are 2580 (31%) and 2258 (27%) more cases of bowel cancer and 692 and 609 (27% and 24%) more deaths in the two lowest socioeconomic quintiles (Q1 and Q2) compared to the highest (Q5). Even when screened, the two lowest quintiles have 1073 and 1232 (70% and 80%) fewer follow-up colonoscopies than those from the highest quintile, contributing to higher case numbers, more advanced CRC cases and deaths. Our modelling of the impact of increasing screening rates and colonoscopy rates of the lower quintiles to match those of Q5 indicated potential increases in adenoma or CRC detected of 445 (101.5%) and 268 (49.2%) in Q1 and Q2 respectively.

The higher deaths and cancer cases predicted in the lower quintiles are unsurprising given differences in screening rates, diagnosis, lifestyle risk factors, treatment and outcomes and access to healthcare services compared with the higher quintiles [38, 39]. The difference in screening rates between Q1 and Q4 and Q5 in SA of approximately 10% contributes to the widening socioeconomic differences in CRC outcomes, and the gap in screening rates appears to be widening. A previous study of NBCSP screening rates in SA using data from 2007–8 found that those from the most deprived quintile had 8% lower participation rates (40%) than the least deprived (48%) [40]. Our findings from 2018–19 data indicate that in the last decade screening rates have increased, but the least deprived have increased their rates of screening about 2% more (51.7%) than the most deprived (41.9%), a difference of 9.8%. The differences in screening rates undoubtedly result in the differences in stage at diagnosis; there were 12% less unscreened patients in Stage 1 and 12% more in Stage 4 than screened patients in the linked dataset. By improving screening rates, detection at earlier stages is more likely.

Overcoming the barriers to screening in disadvantaged groups remains a challenge. Negative beliefs particularly prevalent in this group, such as cancer screening being threatening, difficult to accomplish and not beneficial, need to be countered [41]. Lack of social norms are also predictors of non-participation in cancer screening [41]. Improving health literacy will help patients increase their knowledge and understanding of bowel cancer screening, leading to an increase in screening uptake, a decrease in the social inequalities in screening and potential inequalities in survival [7, 42].

A previous study examining a similar dataset from SA for CRC patients in 2003─2008 found the hazard ratio for death to be 0.75 (95% 0.62─0.91) for the highest socioeconomic quintile, or a 25% reduction in risk of death compared with the lowest [43]. These findings are similar to the mortality differences we found between the higher and lowest quintiles. Previous studies that have modelled the long-term health outcomes of CRC in Australia have not considered socioeconomic differences, making comparison of our results challenging [14, 31, 44–46]. Comparing the outcomes of our study with others is possible when similar costs, outcomes and participation rates and assumptions have been used. Of these five previous studies, four are not directly comparable due to either not accounting for treatment or colonoscopy surveillance costs or using outdated costs of treatment [31, 44–46]. The study by Lew et al [14] is the most comparable using recent cost estimates and a 40% participation rate scenario, comparable to our participation rates of 42% in quintile 1. Lew and colleagues estimated mean life years per person of 15.597, increasing to 15.610 for 60% participation or an increase of 0.08%. Our QALY estimates were 12.86 in Q1, 0.5% lower than Q5 at 12.91, are expected to be lower than the Lew study as ours are adjusted for quality of life, but percentage differences between the quintiles are close to the Lew et al study [14].

A strength of our study is the use of data extracted from a population-based linked dataset containing CRC patients from SA using multiple health and administrative datasets. This included screening status of patients, stage of diagnosis by SEIFA and survival by cancer stage. Unfortunately, not all data was available by SEIFA due to small cell sizes, however national data by SEIFA was used where available. We were unable to obtain more recent data for the South Australian population as this was not publicly available, therefore the number of deaths may be overstated. However, the socioeconomic disparities would likely be relatively very similar [47]. As with all modelling studies there are limitations. The proportions in each stage of cancer extracted from the linked dataset, were assumed to remain constant. This may over or under estimate future outcomes. We did not use sensitivity and specificity rates of FOBTs and colonoscopies, as the reported diagnostic results were used to calculate the probabilities of adenoma, cancer and no disease in the model [17]. However, based on data from the 2006–2010 NBCSP participants, less than 0.1% of those with a negative screen were diagnosed with bowel cancer within 2 years [4]. We did not account for costs or health outcomes of adverse events of colonoscopy therefore these will be underestimated. We also assumed that patients who did not have a follow-up colonoscopy after a positive FOBT moved to and remained in the not screened group, with a higher risk of late-stage cancer. The model assumes the risk of CRC remains stable over the 50 year period.

## Conclusions

At current levels of screening, we see the long-term socioeconomic differences in higher rates of CRC and deaths in lower SEP groups in SA over 50 years. Also apparent are the higher rates of assessing individuals with positive FOBT tests and subsequent early detection of adenomas and cancers in higher SEP quintiles. Applying these results nationally, implementing targeted programs to increase screening and follow up tests in disadvantaged groups is critical in improving CRC outcomes population-wide. Evaluating these programs for their effectiveness and cost-effectiveness needs prioritising. Ultimately this would lead to better cancer-related outcomes amongst disadvantaged groups, leading to improved long-term survival and reductions in the emotional, social and economic burden borne by themselves, their families and the healthcare system.

## Supporting information

S1 Appendix (PDF)

S1 Table (PDF)

## References

[1] Australian Institute of Health and Welfare. Australian Burden of Disease Study: Impact and causes of illness and death in Australia 2015. Australian Burden of Disease series no. 19. Cat. no. BOD 22. Canberra: AIHW; 2019.

[2] Goldsbury DE, Yap S, Weber MF, Veerman L, Rankin N, Banks E, et al. Health services costs for cancer care in Australia: Estimates from the 45 and Up Study. PLOS ONE. 2018;13:7. doi: 10.1371/journal.pone.0201552 30059534 PMC6066250

[3] Australian Institute of Health and Welfare. Cancer in Australia 2019. Cancer series no.119. Cat. no. CAN 123. Canberra: AIHW; 2019.

[4] Australian Institute of Health and Welfare. Analysis of bowel cancer outcomes for the National Bowel Cancer Screening Program: 2018. Cat. no. CAN 113. Canberra: AIHW; 2018.

[5] Todorov K, Wilson C, Sharplin G, Corsini N. Faecal occult blood testing (FOBT)-based colorectal cancer screening trends and predictors of non-use: findings from the South Australian setting and implications for increasing FOBT uptake. Aust Health Rev. 2018;42:1. 28248632 10.1071/AH16126

[6] Sørensen K, Van den Broucke S, Fullam J, Doyle G, Pelikan J, Slonska Z, et al. Health literacy and public health: A systematic review and integration of definitions and models. BMC Public Health. 2012;12:1. 22276600 10.1186/1471-2458-12-80 PMC3292515

[7] Durand M-A, Lamouroux A, Redmond NM, Rotily M, Bourmaud A, Schott A-M, et al. Impact of a health literacy intervention combining general practitioner training and a consumer facing intervention to improve colorectal cancer screening in underserved areas: protocol for a multicentric cluster randomized controlled trial. BMC Public Health. 2021;21:1. 34530800 10.1186/s12889-021-11565-3 PMC8444501

[8] Department of Health. Population Based Screening Framework. Canberra: Commonwealth of Australia; 2018.

[9] Worthington J, Lew J-B, Feletto E, Holden CA, Worthley DL, Miller C, et al. Improving Australian National Bowel Cancer Screening Program outcomes through increased participation and cost-effective investment. PLOS ONE. 2020;15:2. doi: 10.1371/journal.pone.0227899 32012174 PMC6996821

[10] Australian Bureau of Statistics. 3235.0 Regional Population by Age and Sex, Australia. Canberra: ABS; 2020.

[11] Australian Bureau of Statistics. Census of Population and Housing: Socio-Economic Indexes for Areas (SEIFA) Canberra: ABS; 2018 [cited 19 April, 2021]. Available from: https://www.abs.gov.au/ausstats/abs@.nsf/Lookup/by%20Subject/2033.0.55.001~2016~Main%20Features~IEO~22

[12] Australian Bureau of Statistics. 3101.0—Australian Demographic Statistics Canberra: ABS; 2019.

[13] South Australian Cancer Registry, Wellbeing SA. Cancer in South Australia 2017– with projections to 2020. Adelaide: Prevention and Population Health Directorate, Government of South Australia; 2020.

[14] Lew JB, St John DJB, Xu XM, Greuter MJE, Caruana M, Cenin DR, et al. Long-term evaluation of benefits, harms, and cost-effectiveness of the National Bowel Cancer Screening Program in Australia: a modelling study. Lancet Public Health. 2017;2:7. 10.1016/S2468-2667(17)30105-6 29253458

[15] Kuntz KM, Lansdorp-Vogelaar I, Rutter CM, Knudsen AB, van Ballegooijen M, Savarino JE, et al. A systematic comparison of microsimulation models of colorectal cancer: the role of assumptions about adenoma progression. Medical decision making: an international journal of the Society for Medical Decision Making. 2011;31:4. doi: 10.1177/0272989X11408730 21673186 PMC3424513

[16] Stryker SJ, Wolff BG, Culp CE, Libbe SD, Ilstrup DM, MacCarty RL. Natural history of untreated colonic polyps. Gastroenterology. 1987;93:5. doi: 10.1016/0016-5085(87)90563-4 3653628

[17] Australian Institute of Health and Welfare. National Bowel Cancer Screening Program monitoring report 2019. Cancer series no. 125. Cat. no. CAN 125. Canberra: AIHW; 2019.

[18] Australian Institute of Health and Welfare. National cancer screening programs participation data. Supplementary data Canberra: AIHW; 2019 [cited 15 April, 2020]. Available from: https://www.aihw.gov.au/reports/cancer-screening/national-cancer-screening-programs-participation/contents/national-bowel-cancer-screening-program/participation

[19] Australian Institute of Health and Welfare. National Bowel Cancer Screening Program monitoring report 2020. Cancer series no.126. Cat. no. CAN 133. Canberra: AIHW; 2020.

[20] Packaging Stanley. 200pcs 120mm x 180mm Bubble Padded Mailer Envelope Melbourne 2020 [cited 28 April, 2020]. Available from: https://www.stanleypackaging.com.au/product/bubble-padded-bag-mailer-envelopes-120mmx180mm/?gclid=Cj0KCQjwhZr1BRCLARIsALjRVQPGXM1mhsFfmU5m_Xx0_H8eUrrveTmzRXprWEyy9Cpca_z_6R8Y6VUaAjWhEALw_wcB

[21] Australia Post. Calculate postage and delivery 2020 [cited 28 April, 2020]. Available from: https://auspost.com.au/parcels-mail/calculate-postage-delivery-times/#/

[22] Australian Government Department of Health. Medicare Benefits Schedule Book. Canberra Commonwealth of Australia; 2018.

[23] Independent Hospital Pricing Authority. National Hospital Cost Data Collection Report, Public Sector, Round 22 (Financial year 2017–18). Appendix Tables. Sydney: IHPA; 2020.

[24] Ananda S, Kosmider S, Tran B, Field K, Jones I, Skinner I, et al. The rapidly escalating cost of treating colorectal cancer in Australia. Asia Pac J Clin Oncol. 2016;12:1. 25866889 10.1111/ajco.12350

[25] Australian Institute of Health and Welfare. Health expenditure Australia 2017–18 Supplementary tables. Canberra, Australia AIHW; 2019.

[26] Australian Government Department of Health and Aged Care. Guidelines for preparing a submission to the Pharmaceutical Benefits Advisory Committee (version 5.0) Canberra, Australia 2016 [cited 5 November, 2021]. Available from: https://pbac.pbs.gov.au/

[27] Davis NC, Newland RC. Terminology and classification of colorectal adenocarcinoma: The Australian clinico-pathological staging system. Australian and New Zealand Journal of Surgery. 1983;53:3. 6309132 10.1111/j.1445-2197.1983.tb02430.x

[28] Chan C, Chapuis P. Cancer Council Australia’s Clinical Guidelines. Selection of a clinicopathological staging system Sydney, Australia: Cancer Council Australia; 2017 [cited 28 October, 2022]. Available from: https://wiki.cancer.org.au/australia/Guidelines:Colorectal_cancer/Selection_of_a_clinicopathological_staging_system

[29] Australian Bureau of Statistics. 2033055001 Socio-Economic Indexes for Australia SEIFA 2016. Table 1 Statistical Area Level 2 SEIFA Summary 2016. Canberra: ABS; 2018.

[30] Frazier AL, Colditz GA, Fuchs CS, Kuntz KM. Cost-effectiveness of screening for colorectal cancer in the general population. Jama. 2000;284:15. doi: 10.1001/jama.284.15.1954 11035892

[31] Bishop J, Glass P, Tracey E, Hardy M, Warner K, Makino K, et al. Health Economics Review of Bowel Cancer Screening in Australia. Sydney, Australia: Cancer Institute NSW Monograph; 2008.

[32] Australian Institute of Health and Welfare. Cancer Data in Australia; Australian Cancer Incidence and Mortality (ACIM) books: colorectal cancer. Canberra: AIHW; 2018.

[33] Australian Bureau of Statistics. Life Tables, States, Territories and Australia, 2016–2018. Canberra: ABS; 2019.

[34] Drummond MF, Sculpher MJ, Torrance GW, O’Brien B, Stoddart G. Methods for the economic evaluation of health care programmes. 4th ed. Oxford: Oxford University Press; 2015.

[35] Neumann PJ, Sanders GD, Russell LB, Siegel JE, Ganiats TG. Cost-effectiveness in health and medicine. 2nd Edition ed: Oxford University Press; 2016.

[36] Djalalov S, Rabeneck L, Tomlinson G, Bremner KE, Hilsden R, Hoch JS. A Review and Meta-analysis of Colorectal Cancer Utilities. Medical decision making: an international journal of the Society for Medical Decision Making. 2014;34:6. doi: 10.1177/0272989X14536779 24903121

[37] Hawthorne G, Korn S, Richardson J. Population norms for the AQoL derived from the 2007 Australian National Survey of Mental Health and Wellbeing. Aust N Z J Public Health. 2013;37:1. doi: 10.1111/1753-6405.12004 23379800

[38] Aarts MJ, Lemmens VE, Louwman MW, Kunst AE, Coebergh JW. Socioeconomic status and changing inequalities in colorectal cancer? A review of the associations with risk, treatment and outcome. Eur J Cancer. 2010;46:15. doi: 10.1016/j.ejca.2010.04.026 20570136

[39] Palmer RC, Schneider EC. Social disparities across the continuum of colorectal cancer: a systematic review. Cancer Causes Control. 2005;16:1. doi: 10.1007/s10552-004-1253-3 15750858

[40] Martini A, Javanparast S, Ward PR, Baratiny G, Gill T, Cole S, et al. Colorectal cancer screening in rural and remote areas: analysis of the National Bowel Cancer Screening Program data for South Australia. Rural Remote Health. 2011;11:2. 21585228

[41] von Wagner C, Good A, Whitaker KL, Wardle J. Psychosocial determinants of socioeconomic inequalities in cancer screening participation: a conceptual framework. Epidemiol Rev. 2011;33. doi: 10.1093/epirev/mxq018 21586673

[42] Berkman ND, Sheridan SL, Donahue KE, Halpern DJ, Crotty K. Low Health Literacy and Health Outcomes: An Updated Systematic Review. Annals of Internal Medicine. 2011;155:2. doi: 10.7326/0003-4819-155-2-201107190-00005 21768583

[43] Beckmann KR, Bennett A, Young GP, Cole SR, Joshi R, Adams J, et al. Sociodemographic disparities in survival from colorectal cancer in South Australia: a population-wide data linkage study. BMC health services research. 2016;16. 26792195 10.1186/s12913-016-1263-3 PMC4721049

[44] Pignone MP, Flitcroft KL, Howard K, Trevena LJ, Salkeld GP, St John DJ. Costs and cost-effectiveness of full implementation of a biennial faecal occult blood test screening program for bowel cancer in Australia. The Medical journal of Australia. 2011;194:4. 21401458 10.5694/j.1326-5377.2011.tb03766.x PMC3136747

[45] Stone CA, Carter RC, Vos T, John JS. Colorectal cancer screening in Australia: an economic evaluation of a potential biennial screening program using faecal occult blood tests. Aust N Z J Public Health. 2004;28:3. 15707175 10.1111/j.1467-842x.2004.tb00707.x

[46] O’Leary BA, Olynyk JK, Neville AM, Platell CF. Cost-effectiveness of colorectal cancer screening: comparison of community-based flexible sigmoidoscopy with fecal occult blood testing and colonoscopy. J Gastroenterol Hepatol. 2004;19:1. 14675241 10.1111/j.1440-1746.2004.03177.x

[47] Stanbury JF, Baade PD, Yu Y, Yu XQ. Cancer survival in New South Wales, Australia: socioeconomic disparities remain despite overall improvements. BMC Cancer. 2016;16:1. doi: 10.1186/s12885-016-2065-z 26832359 PMC4736306

