# Supplementary material for: Long-term benefits for lower socioeconomic groups by improving bowel screening participation in South Australia: A modelling study
Source: PLoS One. 2022 Dec 21;17(12):e0279177. doi: 10.1371/journal.pone.0279177 (PMC9770333; doi:10.1371/journal.pone.0279177)
Supplement: S1 Appendix — (PDF) [file pone.0279177.s001.pdf]

S1 Appendix

Model overview

The Priority Population Microsimulation Colorectal Cancer (PRISM-CRC) model is a Markov microsimulation model, consisting of five separate Markov nodes for each socioeconomic quintile. Based on the age distribution in the South Australian (SA) population, individuals are simulated through yearly transitions. The model uses Monte Carlo simulation to predict the probability of different events. These events include death, adenoma incidence, cancer incidence and transitions from one stage of cancer to another.

The model consists of three parts 1) natural cancer development 2) screening and 3) colonoscopy surveillance.

Natural Cancer development

The model simulates the natural development of colorectal cancer. It is assumed that colorectal cancer develops through the adenoma-carcinoma pathway. Adenomas develop according to an age specific incidence rate of low risk <10mm or high risk >10mm. Adenomas are either non-progressive, i.e. will not develop into cancer, or are progressive adenomas which become malignant and transform into Stage 1 cancer. It was assumed that 20 years is the time from progressive adenoma to clinical diagnosis [1]. A non-symptomatic cancer can remain undiagnosed or may be symptomatically diagnosed. This can include cancers of individuals who do not participate in screening, or who did not have a follow-up colonoscopy after a positive immunochemical faecal occult blood test FOBT test or had an inaccurate negative FOBT test. If the cancer remains undiagnosed, it can progress from Stage 1 through each stage to Stage 4. Survival after diagnosis depends on the cancer stage at diagnosis and the time since diagnosis.

Screening

The second part of the model is the bowel cancer screening which disrupts the natural development of cancer. The model simulates the National Bowel Cancer Screening Program (NBCSP) from 2019, which became a biennial screening program for ages 50-74 years. Immunochemical Faecal Occult Blood Test (FOBT) are assumed to be sent to all eligible participants to complete at home. Based on population numbers of SA from the Australian Bureau of Statistics, the number of participants in the cohort aged 50-74 years was 513,000 or 102,600 per socioeconomic quintile [1]. Tests that were returned were assumed to be completed correctly. An individual returning a negative test would re-enter the screening program and be sent an FOBT kit when they were eligible again. An individual returning a positive test would visit a general practitioner for referral for a colonoscopy procedure.

The three possible outcomes following colonoscopy are adenoma detected, cancer detected or no disease. It was assumed that no adverse events occurred following a colonoscopy. If the individual decides not to have a colonoscopy, they will move to the natural cancer development part of the model and cancer would be detected via symptoms.

Colonoscopy Surveillance

If an adenoma is detected via colonoscopy as part of the screening program, the individual is moved to the colonoscopy surveillance section of the model. Individuals visit a general practitioner every five years to obtain a referral for the colonoscopy. Adenomas detected are removed. Colonoscopy surveillance is assumed to take place until age 75 years. It was assumed that no adverse events occurred following a colonoscopy.

Figure 2 Screening pathway and outcomes

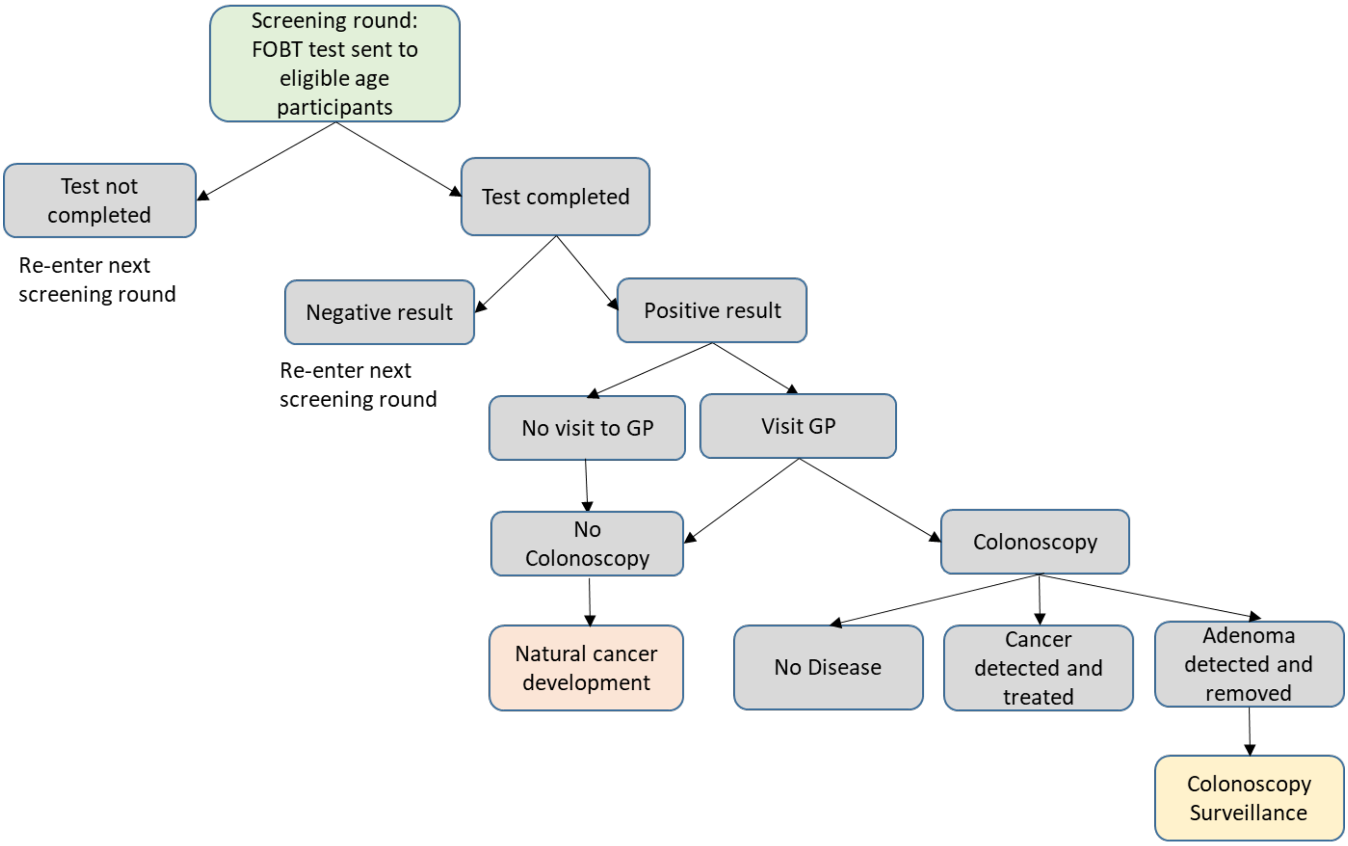

References

1. Loeve F, Brown ML, Boer R, van Ballegooijen M, van Oortmarssen GJ, Habbema JDF. Endoscopic Colorectal Cancer Screening: a Cost-Saving Analysis. JNCI: Journal of the National Cancer Institute. 2000;92:7.
