# Supplementary material for: Long-term benefits for lower socioeconomic groups by improving bowel screening participation in South Australia: A modelling study
Source: PLoS One. 2022 Dec 21;17(12):e0279177. doi: 10.1371/journal.pone.0279177 (PMC9770333; doi:10.1371/journal.pone.0279177)
Supplement: S1 Table — (PDF) [file pone.0279177.s002.pdf]

S1 Table. Distribution of SA population start age.

Age (years)	Proportion
50	0.0446
51	0.0448
52	0.046
53	0.0468
54	0.0462
55	0.0473
56	0.0458
57	0.0453
58	0.0439
59	0.0433
60	0.0427
61	0.0415
62	0.0408
63	0.0409
64	0.0398
65	0.0397
66	0.039
67	0.0379
68	0.0372
69	0.0402
70	0.0328
71	0.0314
72	0.0294
73	0.0269
74	0.0258

Source: ABS [1]

Reference

1. Australian Bureau of Statistics. 3235.0 Regional Population by Age and Sex, Australia. Canberra: ABS; 2020.
